# Surgery of the Gall-Bladder and Ducts

**Published:** 1898-01

**Authors:** J. McFadden Gaston

**Affiliations:** Atlanta, Ga.


### SURGERY OF THE GALL-BLADDER AND DUCTS.*

By J. McFADDEN GASTON, M.D.,

Atlanta, Ga.

*Continued from the December number.*

It is not always practicable to find all the stones at the outset, and those not encountered at the operation have escaped subsequently or have been extracted by the operators. This shows the propriety of exploring the cavity of the gall-bladder and the cystic duct on dressing the wound, during the first week after an operation.

The first case of Parkes is very instructive touching this point, and in his graphic account of it on January 27th, 1885, I was informed that an exploration of the cavity of the gall-bladder on the occasion of the operation failed to find any stones in the bladder. On the second day the dressings were charged with a profuse flow of greenish-colored fluid from fistula. On the fourth day again dressed; while probing the bladder this day he detected and removed two stones. On the seventh day he found and removed five stones, one as large as the end of the finger. None were found subsequently.

He was satisfied that a contraction of the common duct existed, rendering it too small in caliber to carry off the biliary secretion, and that for the time being the fistula would have to be maintained open.

Had this been evident on the occasion of passing the sound, Parkes might doubtless have adopted the suggestion at page 380, in the October number of *Gaillard's Journal,* that "in case any communication with the duodenum exist, however small it may be, dilatation of the ductus choledochus should be attempted."

Notwithstanding the assurance of Parkes that his finger in the abdominal cavity guided the sound through the cystic and common ducts into the duodenum, and the statement of Sims in the autopsy

* Abstract of paper presented to the Southern Surgical and Gynecological Association, at its meeting on November 9, 1897, in St. Louis, Mo.

of his case, that "Ganal was able to pass a probe from the gall-bladder through the ductus communis choledochus into the duodenum," I observe at page 100 of the *Medical Bulletin* for April, 1885, that Bartholow questions the practicability of catheterization and consequent dilatation of these ducts. While I encountered this difficulty in the post mortem of a dog reported at page 83 of the *Southern Medical Record*, for March, 1885, numerous other examinations were made in dogs, presenting no obstacle to the passage of a sound from the gall-bladder through the cystic and common ducts into the duodenum. While a straight metallic probe may not pass readily from the cystic into the common duct, there is no embarrassment in carrying a curved sound or an elastic bougie from the gall-bladder into the duodenum, and hence in cases of partial constriction of either duct, the successive use of graduated sounds or bougies may be relied upon for dilatation of the passages

With the lights before us, it is clear that an effort should be made to overcome temporary obstructions of the ducts, and if concretions exist which cannot be moved in either direction by pressure from without the canal with the fingers, an effective recourse is found in adopting the expedient of Tait for crushing the masses with padded forceps, and leaving the fragments to be flooded out by the bile. This suits cases not requiring incision of the sac, but he is in favor of establishing a biliary fistula temporarily, and a large fenestrated drainage-tube should be inserted, while the whole abdominal wound is securely closed excepting at the opening for the attachment of the incision in the sac. A compress and bandage should be used to obviate any tendency to ventral hernia after the operation.

Keen also regards the creation of a biliary fistula as the better practice, yet he holds that in case a probe could be passed into the duodenum with a reasonable assurance that all stones have been removed, closure of the gall-bladder by Gély's suture or otherwise might be done with propriety.

In comparing the different measures adopted by surgeons for the relief of a distended gall-bladder, we shall find that their rela–

tive advantages depend upon the greater or less probability of restoring the bile to the intestines.

Whatever may be the properties of the bile, it would seem to have been designed for a useful purpose by admixture with the chyme soon after leaving the stomach, and in combination with the pancreatic secretion enables the lacteals to take up the nutritive elements of the food. While serving as an antiseptic, it also promotes the peristaltic action of the intestines.

If a stone remain in the cystic duct the sac will be refilled with its own secretion, and if in the common duct by bile; but should all the stones be removed and the ducts be pervious, the fistula will soon close under the use of drainage through the duct into the duodenum.

Should the bile be certainly restored to its natural channel on the occasion of an operation, the closure of the incision in the sac and dropping it into the abdomen, with complete suture of the external wound, are advisable.

Besides the case of Bobbs, in which only one stitch was used to close the incised sac, those of Courvoisier and Bernays, operated on by this method, have proved entirely satisfactory. Even the fatal case of Meredith, in which the incision of the gall-bladder was closed with the continued suture and dropped into the abdominal cavity, proved the feasibility of immediate closure under favorable conditions. In the succinct report of it forwarded to me February 11th, 1885, the author states that this patient died in forty-eight hours with suppression of urine. "At the autopsy the incision in the gall-bladder was found securely closed, and quite impervious to fluid."

The case of Dr. Courvoisier died from pneumonia two and a half months subsequent to the operation, and the autopsy showed a perfect union from the suture of the incised wall of the gall-bladder.

There are some cases, in which complications exist, that preclude the completion of an operation which has been undertaken, such as extensive disorganization, the agglutination of tissues or the evidences of malignant disease. Of the last Senn reported an interesting case to me on February 11th, 1885, with the remark, that it is sometimes more important to report unfavorable than favorable cases. A parallel case to this has been reported since in Germany, and though, as Senn states, primary cancer of the bile-duct is an exceedingly rare occurrence, scirrhus of the pancreas and pylorus often involves it in the disease.

Some interesting and instructive notices of the great advances made in operative procedures for disorders of the gall-bladder, appeared in the numbers of the *Medical News* of Philadelphia for December 20th, 1884, and February 14th, 1885.

When the outlet of the common bile-duct is permanently closed, and the bile finds its way from the hepatic duct through the cystic duct into the gall-bladder, the most philosophic proceeding is to effect a direct passage of the bile from the gall-bladder into the duodenum.

In my first series of experiments upon dogs for testing this union of the gall-bladder and duodenum a circular row of catgut continuous suture attached the serous membranes around an elastic ligature connecting the two walls. This was done under the impression that it was requisite to excite adhesive inflammation, by which the surfaces should become agglutinated. But it was productive of undue disturbance, and in the last case of that series it was observed that sufficient inflammatory action ensued from the insertion of a single stitch of silk thread to unite the walls of the gall-bladder and duodenum.

With the further observation that this loop cut an opening between the two cavities, in case an incision be made into the sac for exploration, it may be closed by Lembert's suture separate from the parietes of the abdomen; and the external wound may then be sutured throughout without any provision for drainage, as the bile passes very soon through the apertures of the thread and ultimately through the fistula.

With a view to diagnosticate distension of the gall-bladder, all tumors connected with the liver should be examined with care. A peculiarly complicated case of laparotomy is reported by Bauer in the *St. Louis Medical and Surgical Journal* for June, 1886, in the person of a mechanic aged forty.

This case was calculated to mislead the operator, in view of the fact that "immediately below the right hypochondrium there was a tumor obviously connected with the liver, since it moved contemporaneously with that organ. It presented a semi-globular form, about three inches in diameter, was smooth and elastic to the touch, and withal very tender." The author leaves us entirely in doubt as to his preconceived view by saying that although he had formed some diagnostic views on the nature of the tumor, believing that laparotomy is the only trustworthy diagnostic method, he proceeded to operate. (Query—What does an analysis of the facts prove?) [Abscess of Liver.]

A paper read by Keen before the Philadelphia Academy of Surgery November 2, 1885, presents some interesting points bearing upon the relations of the duodenum to the gall-bladder and liver.

An interesting case of fatal obstruction of the ileo-cecal valve by a gall-stone was reported by Dr. Russell H. Johnson in the *Medical News* of June 27, 1885. The author states that "the facts of the case warrant the conclusion that the gall-stone under consideration escaped from the gall-bladder by means of a fistulous tract connecting it with the duodenum," and remarks further that "autopsic examinations (especially those made by Murchison) have in many instances shown the presence of these fistulas and lead us to infer that it is not a very unusual occurrence."

A very instructive report to the New York Surgical Society, at its meeting of April 28, 1885, by Dr. Peters, detailing the results of an autopsy, illustrates strikingly this ulcerative tendency from an occlusion of the gall-bladder and the accompanying adhesions surrounding the perforation into the duodenum by which nature provides an outlet from the gall-bladder into the duodenal canal.

It is evident that the slow progress of such cases at the outset, and the gradual concretion of hard masses in the gall-bladder, favor the development of a subacute form of inflammation, which leads to suppuration in the sac and ulceration of adjacent tissues. The inflammatory action which is sometimes set up in the tissues of the gall-bladder seems to point to relief in the direction of the duodenum, and an autopsic observation of the ulcerative process by which the contents of the gall-bladder passed into this canal first directed my attention to the practicability of effecting a direct communication between these two organs.

No measure apart from that of Von Winiwarter had been adopted previously with a view to convey the bile from the gall-bladder into the intestinal canal. In his case the gall-bladder was first united to the colon, and after the lapse of some time he established a fistula between the sac and the small intestine by uniting them with stitches, suturing the intestine to the abdominal wall, and opening the gut on the fifth day, puncturing the opposed surfaces through the incised intestine, and finally closing the latter with sutures. This operation of Winiwarter was intended to remedy the occlusion of the common bile-duct by effecting an artificial communication with the intestine for the passage of the bile, and while it failed to realize the advantages expected from a cholecysto-duodenal outlet, the partial success of this procedure shows the tolerance of similar measures by the respective structures involved. No complications arose in Winiwarter's case from the coils of intestine, the constriction of its lumen, or the regurgitation of its contents into the gall-bladder; and much less are such difficulties likely to occur in the attachment of the sac to the duodenum.

The practical discernment of Harley led him to the conclusion that the triumph of operative surgery would be to establish an artificial fistula between the gall-bladder and the duodenum. For then not only would the pent-up bile be removed, but the disturbance arising from the non-admittance of bile into the intestines likewise be at the same time overcome. I am not quite sure, he remarks, if, in these days of antiseptic surgery, the operation is not quite practicable; for I can see no reason why the adjacent surfaces of the gall-bladder and duodenum should not be eroded by potassa-fusa and speedily stitched together.

The principle of cholecysto-duodenal communication seems to have been grasped in this suggestion for creating a fistulous opening; but the process of accomplishing it presents objectionable features, which do not hold against the plan of uniting the two walls by a loop of suture which shall induce an amount of adhesive inflammation sufficient for the union of their outer surfaces, with a certainty of cutting through the adjoining tissues and effecting a passage for the bile from the gall-bladder into the duodenum.

Harley is justly entitled to priority for the publication of this suggestion "to establish an artificial fistula between the gall-bladder and duodenum," as his book on Diseases of the Liver, which contains it, appeared prior to the publication of my article in *Gaillard's Journal* on "Obstruction of the Gall-duct and Its Bad Consequences, with Remedial Operation Suggested." But it is due to myself to state that his work had not been seen, nor was I aware of his suggestion when my paper was published. In like manner it may be stated that I did not know of the operation of Winiwarter, though it was recorded in a foreign journal in 1882, and published in the October number of the *American Journal of the Medical Sciences* for 1884.

But, putting aside the question of priority, it is proper to note the difference in a predetermined recourse to the ligation for opening a communication from the gall-bladder into the duodenum, which shall at the same time induce a firm union of their outer surfaces, from an incidental attachment of the sac to the large or small intestine, as done in his case. It must be perceived that his mode of proceeding has quite a dissimilar effect from the operation that was suggested by Harley, or from that process which I adopted afterwards for establishing the flow of the bile into the duodenum, which McGraw has indorsed.

The close proximity of the duodenum to the lower and under surface of the distended gall-bladder, with more or less thickening of its wall, affords favorable conditions for this operation; and it is evident that greater tolerance of the textures for surgical interference is presented after the disorders connected with biliary obstruction have diminished the tendency to active or acute inflammation.

A class of operations calls for attention in connection with impaction of biliary calculi in the cystic or common ducts which cannot be dislodged by the means already mentioned. The incision of the wall of the duct by the knife or scissors, constituting this operation for the removal of stones, has in most cases been closed afterwards by suture, but when this is impracticable it may be left open and the intervening space should be packed with gauze, leaving the ends protruding at the external wound, to afford drainage and prevent the entrance of bile into the peritoneal cavity.

The various modes of procedure adopted by different operators in effecting cholecystenterostomy include anastomosis of the gall-bladder with the duodenum, the small intestine and the colon, by different processes besides that of union by a stitch of silk thread or the elastic ligature.

The attached surfaces have been sutured, with the cut edges brought together or the division made after nearly completing the circuit. The bone-plates have been employed to approximate the walls of the gall-bladder and intestinal canal, and the Murphy button has latterly been used in most cases for this anastomosis.

There has been a fair measure of success by each of these procedures, and the choice of operators in the future will depend to a considerable extent upon the conditions presented in the individual cases.

